# Equine saliva components during mastication, and in vivo pH changes in the oral biofilm of sound and carious tooth surfaces after sucrose exposure

**DOI:** 10.1186/s13028-020-00518-2

**Published:** 2020-05-23

**Authors:** Torbjörn Lundström, Peter Lingström, Ove Wattle, Anette Carlén, Dowen Birkhed

**Affiliations:** 1 Animal Dental Clinic, Västra Husby, 60596 Söderköping, Sweden; 2 Department of Cariology, Sahlgrenska Academy, University of Gothenburg, Box 450, 405 30 Gothenburg, Sweden; 3 Division of Diagnostics and Large Animal Clinical Sciences, Department of Clinical Sciences, Swedish University of Agricultural Sciences, 75007 Uppsala, Sweden; 4 Department of Oral Microbiology and Immunology, Institute of Odontology, Sahlgrenska Academy Sweden University of Gothenburg, Box 450, 405 30 Gothenburg, Sweden; 5 Fersens väg 14B, 211 42 Malmö, Sweden

**Keywords:** Caries, Electrolytes, Equine, Horse, pH drop, Saliva, Sucrose, Teeth, Urea

## Abstract

**Background:**

The role of saliva composition and dietary sugar in development of infundibular caries in equine cheek teeth is not fully understood. This study analysed electrolyte and urea concentrations in saliva in relation to different forage and measured pH changes after sucrose application in vivo in sound and carious cheek teeth.

**Results:**

Forage type had no effect on the equine saliva electrolyte concentrations, which varied considerably both intra- and inter-individually. Chewing resulted in increased values for all electrolytes except bicarbonate. Compared with stimulated human saliva, horse saliva after mastication, contained higher amounts of potassium, calcium and bicarbonate, and less phosphate. The in vivo pH measurements showed a lower resting pH and a more pronounced pH drop after sucrose application in carious teeth compared to sound teeth.

**Conclusions:**

No large differences were found between the composition of equine saliva and human saliva. A more pronounced acidogenicity was found for the carious than sound teeth. Thus, the caries process in equine cheek teeth seems to follow the same pattern as in human teeth, caused by acid production by oral microorganisms after sugar consumption.

## Background

During the last 10–20 years, there has been a change in living conditions for most domestic horses in Sweden. The majority of today´s horses and ponies are found in peri-urban areas rather than the countryside, which has led to a change in feeding regime. Overall, the animals spend less time on pasture and are fed more purchased products, such as “all-in-one feed”. This may lead to reduced chewing time and salivary production. Furthermore, horses and ponies are today used mainly for pleasure and competition and not in daily work as in the past. There are indications that these changes in life-style may have had a negative impact on the dental health of horses, e.g. there has been an increased number of cases for treatments of carious lesions at the equine clinic of the University Animal Hospital (Uppsala, Sweden) and the Animal Dental Clinic (Söderköping, Sweden) during the last decades. Equine dental caries also appears to have increased in other countries [1–3]. New knowledge and diagnostic tools have contributed to the attention of caries decay in equine dentistry.

Knowledge about the causes of equine dental caries is limited, but it is believed that the disease process is identical to that seen in humans, i.e. it is caused by factors such as increased sugar consumption and a shift in the bacterial flora to more aciduric and acidogenic microorganisms. A significant difference in the number of acidogenic *Streptococcus devriesei* present on the cheek teeth surfaces in horses with and without infundibular P2 caries has been reported [4]. It is further well known that saliva flow and composition are of major importance for caries protection [5, 6].

Reduced chewing time, resulting in a lower salivary secretion rate and changes in saliva composition, will most likely increase the risk of dental caries in horses. The current study aimed to look at two important aspects of the condition. First by analysing equine whole saliva after chewing different feeds. The second aim was to perform in vivo plaque-pH measurements in horses in order to determine the changes in acidity on different tooth surfaces of the oral cavity after application of sugar and to evaluate whether such changes in pH could be harmful for the dental hard tissue.

## Methods

### Study design and horses

This study was carried out at the Department of Clinical Sciences, Uppsala, Sweden. Totally four horses were included.

### Horse saliva

**Fig. 1:**
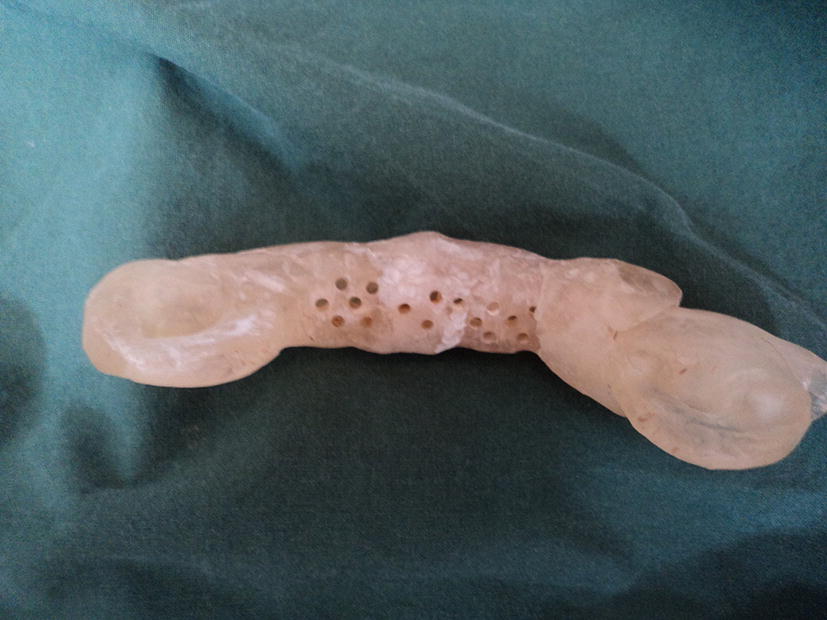
A polymethyl methacrylate (PMMA) mouthpiece with holes specially constructed for the study, adjusted like a bit on a bridle. When the horse is chewing, saliva is collected in the reservoir. After removing the mouthpiece, the saliva can be emptied through a hole on the short side into an Ellerman polystyrene tube

Saliva was collected using a specially made appliance shaped like a mouthpiece of a bit, with holes and a reservoir inside (Fig. 1). The device was applied as a bit on a bridle and in place until the device was filled (1 mL saliva). The collection time was 5 min. Before the samples were taken the horses were starved for minimum 3 h and the oral cavity was thoroughly rinsed with tap-water. Samples were taken on four consecutive days from four horses that each day chewed solely on hay, grass, silage or hay-silage in a randomised crossover study design. Saliva was collected before (0 min) and approximately 50 min after the start of chewing on the forage. The saliva samples were kept frozen at − 20 °C until analysed. After thawing, they were clarified by centrifugation at 1200*g* for 6 min before analysis.

### Human saliva

For reference, stimulated whole saliva was collected from 12 healthy adults (10 females and 2 males; 30–64 years old) into an ice-chilled tube while chewing on a piece of paraffin over a five min period. After collection, the saliva was clarified by centrifugation and kept frozen as described above.

### Electrolytes and urea analysis

Sodium (Na), potassium (K), calcium (Ca), phosphate (P), bicarbonate (HCO_3_) and urea (CH_4_N_2_O) were analysed in the horse and human saliva samples. All analyses were performed in a single session at the Department of Clinical Chemistry and Transfusion Medicine, Sahlgrenska University Hospital, Gothenburg, Sweden. A BM/Hitachi 917 instrument (Boerhringer Mannheim, Indianapolis, IN, USA) was used to analyse Na and K (ion-selective electrode); Ca, urea (light-photospectrometry) and P (enzymatic photospectrometry). Bicarbonate was analysed using an ion-specific electrode and an ABL 505/520 Radiometer (Copenhagen, Denmark). The concentration of the different compounds was expressed in mM.

### Plaque-pH

**Fig. 2:**
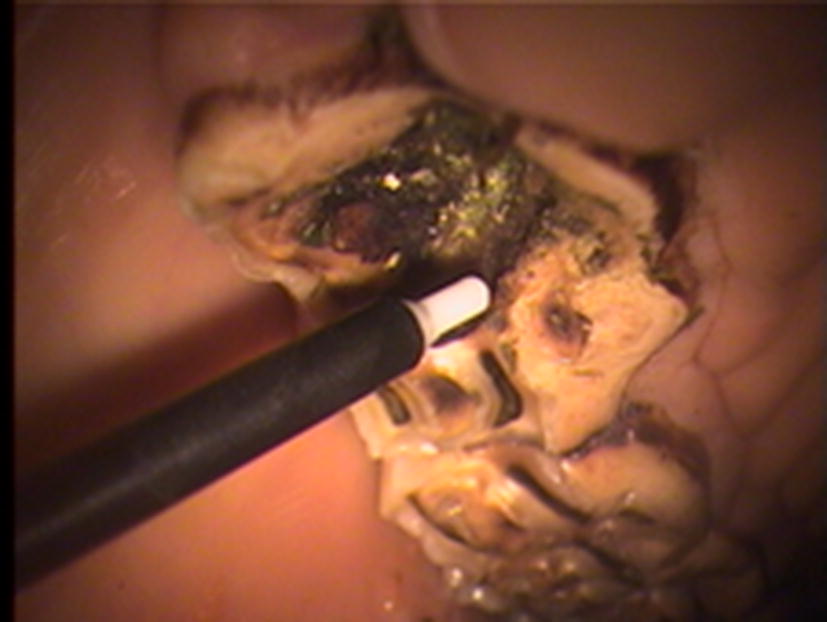
Measurement of plaque pH in situ with the micro-touch method on a carious surface of the infundibulum of the first maxillary premolar tooth. At the end of white plastic holder, there is an ultrathin (0.1 mm in diameter) pH microelectrode, which is connected to reference electrode [7]

Changes in plaque-pH were measured in vivo in the premolar region of the four horses. The measurements were carried out on the occlusal surfaces of healthy teeth, at one site per horse and within the carious lesion of teeth with primary infundibular caries at two sites per horse. Infundibular caries was grade 2 or higher as defined by Lundström et al. [4]. In order to perform the measurements safely and precisely, the horses were sedated with detomidine hydrochloride at 10–20 μg/kg body weight (Domosedan, 10 mg/mL, Orion Pharma AB, Animal Health Sollentuna, Sweden). The head was placed on a support bar to keep the horse in a stable position. An oral speculum (Haussmann) was applied to the front teeth and the oral cavity was rinsed with tap water. Plaque-pH was recorded using the micro-touch method [7] (Fig. 2). The method was a slightly adjusted version of that used in humans, since no cooperation from the animal was possible. Thus, one investigator placed the active electrode on the tooth surface while another held the reference electrode against the soft tissues of the horse during measurements. An iridium microelectrode (Beetrode MEPH-1; W.P. Instruments, New Haven, CT, USA) was used as the active electrode. The electrode was connected to an Orion SA 720 pH/ISE Meter (Orion Research, Boston, MA, USA), to which the reference electrode, an epoxy electrode (DRIREF-5SH; W.P. Instruments) was also connected. After recording resting pH (0 min), approximately 1 mL of 10% sucrose was applied with a pipette to the tooth surface. pH was then measured at three different time points (2, 5 and 10 min) after the application. For each animal, the same sites were used for measurement at all time points. The reference electrode was placed in 3 M KCl solution between measurements. Calibration was made against standard buffer solutions prior to reading of each test value [8].

### Statistical analyses

Due to the small number of individuals tested only descriptive statistics were performed regarding saliva components. Values below the detection limit for sodium and phosphate were set to 5 and 0.05 mM, respectively. Due to the limited number of pH recordings of plaque, only descriptive statistics were performed.

## Results

### Electrolytes and urea in saliva

**Fig. 3:**
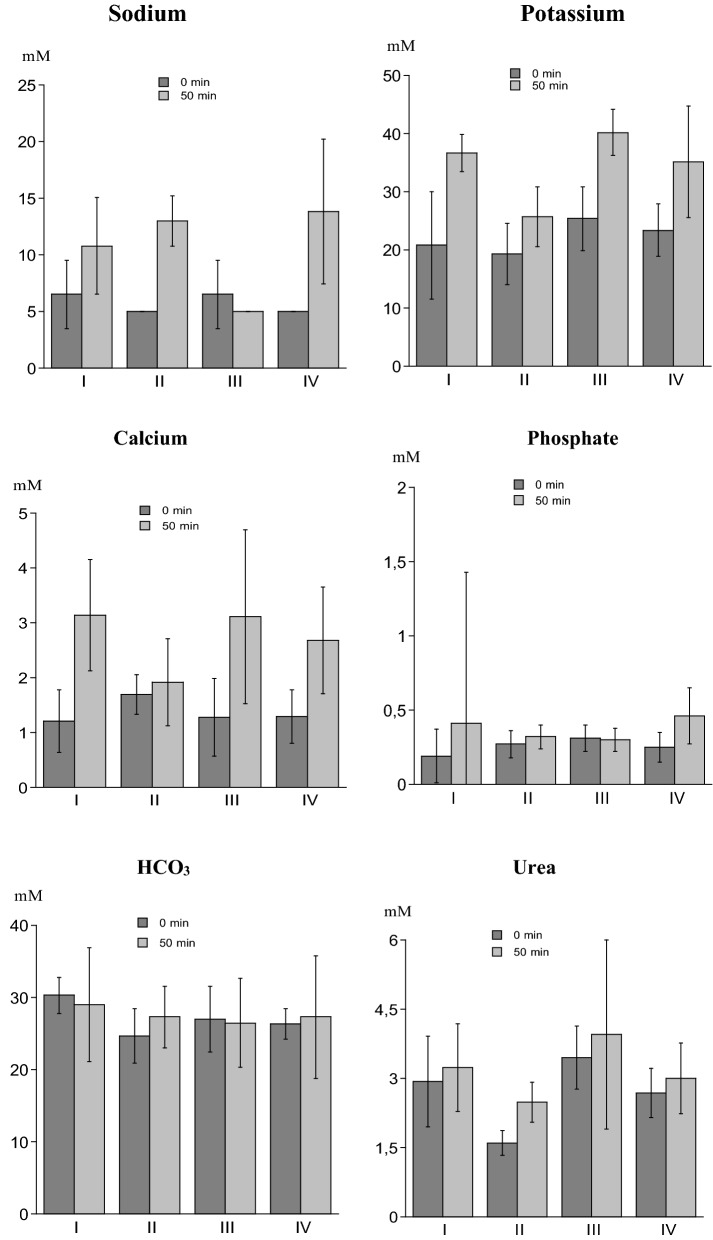
Concentration (mean values ± SD of 4 horses) of sodium, potassium, calcium, phosphate, bicarbonate (HCO_3_) and urea before (0 min) and after 50 min of chewing 4 different forages (I = hay; II = grass; III = silage; IV = hay-silage)

The electrolyte concentration in horse saliva varied considerably both within and between subjects. Differences in the values obtained before and after 50 min of chewing could not be related to type of forage. At baseline (i.e. before chewing), the Na level was low and values above the detection limit (10 mM) were only seen in 2 horses and only in one sample for each horse. Fifty minutes chewing of forage increased the concentrations of all electrolytes, except bicarbonate (Fig. 3). Urea increased numerically by chewing.

Compared to stimulated human saliva, the electrolyte and urea concentrations for horse saliva, both at baseline and after 50 min of chewing, are presented in Table 1.

**Table 1:** Concentrations of electrolytes and urea (mM) in human stimulated saliva (n = 12) and in horse saliva (n = 4; mean of individual means of 4 samples/horse) at baseline (0 min) and after 50 min of forage chewing (mean ± SD and range)

Electrolytesand urea	Saliva		
	Human	Horse 0 min	Horse 50 min
Na^a^	17.3 ± 13.0 (5–51.0)	5.75 ± 0.87 (5.0–6.5)	10.6 ± 4.0 (5.0–13.8)
K	23.5 ± 3.5 (17.8–28.6)	22.2 ± 2.72 (19.3–25.4)	34.4 ± 6.2 (25.7–40.2)
Ca	0.83 ± 0.19 0.52–1.16	1.37 ± 0.22 (1.21–1.70)	2.71 ± 0.57 (1.92–3.14)
P	5.00 ± 2.12 (2.8–9.8)	0.26 ± 0.05 (0.19–0.31)	0.37 ± 0.08 (0.30–0.46)
HCO_3_^−^	21.1 ± 4.0 (17.0–30.0)	27.1 ± 2.39 (24.7–30.3)	27.5 ± 1.06 (26.5–29.0)
Urea	4.66 ± 1.62 (2.3–7.2)	2.67 ± 0.78 (1.60–3.45)	3.17 ± 0.61 (2.48–3.95)

^a^Values < detection limit (10 mM) were set to 5 (3 human samples; 14 out of 16 values at 0 min and 10 out of 16 at 50 min for horses)

### Plaque-pH

**Fig. 4:**
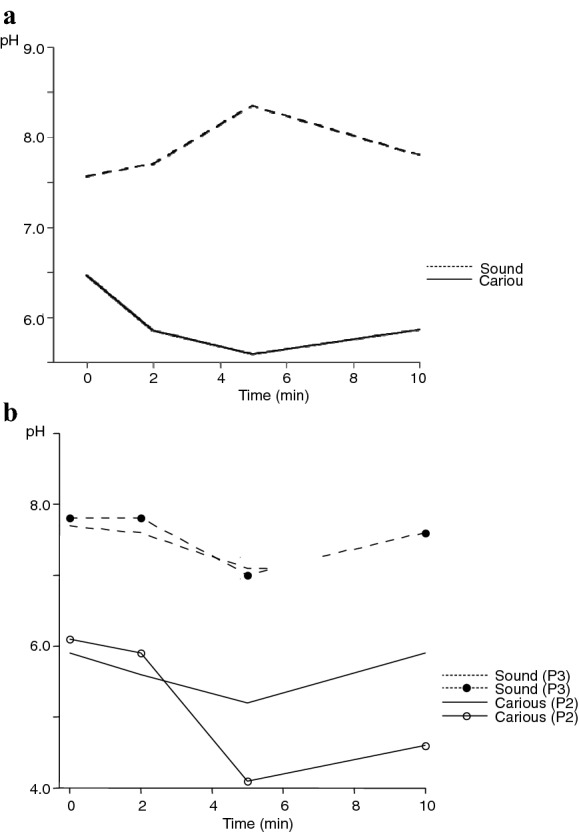
Changes in plaque-pH (mean values) after application of 10% sucrose at **a** 4 sound and 8 carious sites and **b** two sound and two carious sites in one and the same horse

A clear difference in pH response to sucrose was found when comparing the carious and caries-free tooth surfaces with the most pronounced pH drop seen at the carious sites (Fig. 4a). The mean difference expressed as pH units for the different time points varied between 1.1 and 2.8, with a mean difference for all sites and time points of 1.9. No differences were observed with respect to jaw or tooth.

The pH-curves for the carious and sound surfaces differed clearly during the 10-min period but were very similar in shape for the diseased respectively sound sites (Fig. 4b).

## Discussion

The concentrations of electrolytes in human saliva are known to vary in relation to flow rate [9]. Thus, Na and HCO_3_ increase with increasing secretion rate, whereas K, Ca and P are not affected [10]. This differs from the findings of equine saliva where all electrolytes except bicarbonate were increased after 50 min of chewing forage. However, the HCO_3_ concentration was higher in equine saliva than in human saliva and in an earlier study on the parotid saliva in horses, it was found that the bicarbonate depended upon the secretion rate [11]. It is important to remember that horses normally chew for 16–20 h/day and thereby produce between 20 and 80 L of saliva per day so it is therefore difficult to compare human and horse saliva.

The levels of bicarbonate in saliva from horses is of particular interest in relation to the caries process, since bicarbonate is considered to be the most important buffering system in saliva for caries [12]. Together with the cleaning effect of saliva, the buffering of acids in the dental plaque is of major importance in the defence against caries. It is therefore believed that the chewing time, i.e. increased production of saliva in combination with the high concentration of bicarbonate, is an important defence factor against equine infundibular caries. The mechanical cleaning of the occlusal surfaces by attrition also plays an important protective role. Thus, the longer a horse is chewing, the more saliva and the higher amount of bicarbonate is available for neutralising the acid produced by the oral microorganisms.

Urea was of the same magnitude in horse saliva after chewing forage as in the stimulated human saliva. Dental plaque bacteria produce the enzyme urease that hydrolyses urea into ammonia and carbon dioxide [13] which thereby contributes to the buffering of acids in the plaque.

Due to the technical difficulties working on horses in the present study, only a limited number of pH measurements and readings could be performed during a reduced time period. In humans, the pH response is usually monitored for longer periods in order to get a better understanding of both the maximum pH-drop and the pH recovery [7]. However, an interesting observation in the unique in vivo pH-data presented here for horses was that the pH-drop and pH curve seemed to follow the same pattern as in humans when measured with the micro-touch method [8, 12]. This conclusion is based on the application of 10% sucrose, but it is believed that other fermentable carbohydrates, such as fructose and glucose, would also have resulted in a similar pH drop, as shown for humans [14]. Starch is also considered to have a high caries potential in humans because of the high level of salivary alpha-amylase [15], however in horses only a small amount of amylase is present in saliva [16].

The “critical pH” of dental hard tissues (enamel, dentine and cementum), i.e. the pH at which the hydroxyapatite crystals start to dissolve, is an important factor for the demineralisation process and thereby for development of dental caries in humans [17]. There seems to be no major difference between the mineral content of human and horse dental hard tissue with respect to the caries process [18]. However, lesions in equine teeth are markedly different compared to humans [19].

The critical pH of cementum is considered to be around 6.5, while it is around 5.5 for enamel and 6.2 for dentine [14, 19]. It should be mentioned that the critical pH for cementum has been debated and seems to be a complicated issue [20]. The crown of the tooth is covered by enamel in humans, while all three hard tissues (enamel, dentin and cementum) of an equine hypsodont tooth are exposed on the occlusal surface. Caries normally starts in the cementum of the equine tooth. The equine tooth may therefore overall be considered to have a higher critical pH than the human tooth, why human and equine caries cannot be compared on equal terms. Further studies on differences in composition of equine saliva both in healthy horses and horses with caries needs to be carried out to increase the understanding of equine dental caries.

## Conclusions

No major differences in composition were generally found when comparing electrolytes and urea in equine and human saliva. Type of forage had no effect on salivary composition. In vivo pH measurements on sound and carious tooth surfaces showed a reduction in plaque-pH after sucrose exposure. These findings indicate that the caries process in the biofilm of equine cheek teeth follows the same pattern as in human teeth i.e. caries in the horse is caused by acid production by oral microorganisms after sugar consumption.

## Data Availability

The datasets used and/or analyzed during the current study are available from the corresponding author on reasonable request.
